# Metabolomic and transcriptomic analyses reveal the mechanism of sweet-acidic taste formation during pineapple fruit development

**DOI:** 10.3389/fpls.2022.971506

**Published:** 2022-09-08

**Authors:** Yuyao Gao, Yanli Yao, Xin Chen, Jianyang Wu, Qingsong Wu, Shenghui Liu, Anping Guo, Xiumei Zhang

**Affiliations:** ^1^College of Tropical Crops, Hainan University, Haikou, China; ^2^Key Laboratory of Ministry of Agriculture for Tropical Fruit Biology, South Subtropical Crop Research Institute, Chinese Academy of Tropical Agricultural Sciences, Zhanjiang, China; ^3^Taixing Institute of Agricultural Sciences, Taixing, China; ^4^Department of Science Education, Zhanjiang Preschool Education College, Zhanjiang, China; ^5^Sanya Research Institute, Chinese Academy of Tropical Agricultural Sciences, Sanya, China

**Keywords:** *Ananas comosus*, fruit quality, sucrose, citric acid, metabolic genes, transporter genes

## Abstract

Pineapple (*Ananas comosus* L.) is one of the most valuable subtropical fruit crop in the world. The sweet-acidic taste of the pineapple fruits is a major contributor to the characteristic of fruit quality, but its formation mechanism remains elusive. Here, targeted metabolomic and transcriptomic analyses were performed during the fruit developmental stages in two pineapple cultivars (“Comte de Paris” and “MD-2”) to gain a global view of the metabolism and transport pathways involved in sugar and organic acid accumulation. Assessment of the levels of different sugar and acid components during fruit development revealed that the predominant sugar and organic acid in mature fruits of both cultivars was sucrose and citric acid, respectively. Weighted gene coexpression network analysis of metabolic phenotypes and gene expression profiling enabled the identification of 21 genes associated with sucrose accumulation and 19 genes associated with citric acid accumulation. The coordinated interaction of the 21 genes correlated with sucrose irreversible hydrolysis, resynthesis, and transport could be responsible for sucrose accumulation in pineapple fruit. In addition, citric acid accumulation might be controlled by the coordinated interaction of the pyruvate-to-acetyl-CoA-to-citrate pathway, gamma-aminobutyric acid pathway, and tonoplast proton pumps in pineapple. These results provide deep insights into the metabolic regulation of sweetness and acidity in pineapple.

## Introduction

Among the tropical fruits, pineapple (*Ananas comosus* L.) has a production volume of about 25 million metric tons and ranks third in the world after banana and citrus (Ali et al., 2020; Ikram et al., 2020). The useful fruit is processed into value-added compounds because of its pleasant flavor and high abundance of biologically active substances such as vitamins and phenolic and carotenoid compounds (Léchaudel et al., 2018; Ali et al., 2020). China is an important pineapple-producing country, and “Comte de Paris,” the dominant cultivar, accounts for more than 90% of the total pineapple cultivation area (Lu et al., 2014; Zhang et al., 2015). However, because of intensive cultivation focused on yield, commercially produced pineapple fruit seems to have lost its distinct sweet-acidic taste and fruity aroma much like other fruit crops (Klee and Tieman, 2018). Thus, understanding the quality-related metabolic characteristics and genetic basis in pineapple fruit is essential to performing molecular breeding targeting flavor-modulating genes that can contribute to producing a healthy, favorite fruit for consumers.

Sweetness is an important determinant of fruit organoleptic quality, and its intensity is determined by the content and composition of soluble sugars, such as sucrose (Suc), and hexoses, including glucose (Glc) and fructose (Fru) (Vimolmangkang et al., 2016; Klee and Tieman, 2018). Sugar accumulation in fruit is an intricate process that mobilizes the long-distance transport of photoassimilates by sieve element–companion cell complexes of the phloem to sink cells for unloading by symplastic and/or apoplastic pathways (Li et al., 2012; Vimolmangkang et al., 2016). Suc enters the storage parenchyma cells by Suc transporters (SUTs) or hexose transporters after hydrolysis by cell wall invertases (Li et al., 2012). In parenchyma cells, sugar metabolism in the cytoplasm comprises an elaborate system termed the Suc-Suc cycle involving various enzymes, including Suc synthase (SUSY), Suc phosphate synthase (SPS), hexokinase (HK), and phosphofructokinase (PFK) (Li et al., 2012; Aslam et al., 2019). In the vacuole, imported Suc can be irreversibly hydrolyzed to hexose by vacuolar acid invertase (VINV) (Vimolmangkang et al., 2016). In addition to sugar metabolism, sugar accumulation in fruits is regulated by transmembrane transporters. Recently, key sugar transporters contributing to the distribution of sugars in fruit have also been identified, such as the monosaccharide transporter-like (MST), sugars will eventually be exported transporter (SWEET), and the Glc exporter early response to dehydration like 6 (ERDL6) (Wen et al., 2022).

Acidity is also a crucial contributor to fruit taste quality, and its perception depends on organic acid content and composition (Etienne et al., 2013; Klee and Tieman, 2018). The predominant organic acids in most fruits are citrate and malate, which accumulate by complex processes, including synthesis, transport, and degradation or utilization (Zheng et al., 2020; Huang et al., 2021). In the tricarboxylic acid (TCA) cycle, important enzymes such as citrate synthase (CS), aconitase (ACO), malate dehydrogenase (MDH), and isocitrate dehydrogenase (ICDH) play an essential role in citrate and malate metabolism in fruits. In the cytosol, citrate can be degraded or utilized by the gamma-aminobutyric acid (GABA) or acetyl-CoA pathways that affect fruit acidity (Zheng et al., 2020). In addition to organic acid metabolism, acid accumulation is governed by the transport of organic acids from the cytosol to the vacuole. Recently, vacuolar transporters and ion channels and carriers have been reported to play a major role in controlling fruit acidity; for example, the tonoplast proton pumps such as vacuolar-type (V-ATPase, V-PPase) and p-type ATPases, and the channels of aluminum-activated malate transporters (ALMTs) (Huang et al., 2021). In addition to acidic acids and other organic acid components, chlorogenic acid is an important phenolic acid determining the antioxidant capacity and edible value of fruit crops (Chen et al., 2020).

Pineapple is categorized as a non-climacteric tropical fruit and experiences complex biochemical changes, such as variations in the metabolism of sugar, organic acids, and phenolic compounds during fruit development (Saradhuldhat and Paull, 2007; Zhang et al., 2012; Léchaudel et al., 2018). An assessment of sugars and organic acid levels in mature fruits of 26 pineapple cultivars by high-performance liquid chromatography showed that the predominant sugar was Suc, followed by Glc and Fru, and the prevailing acid was citric acid, accompanied by malic and quinic acids (Lu et al., 2014). Sugars and organic acids are not only important sources of fruit attributes, such as sweetness, acidity, and antioxidants, but also play a key role in regulating plant development, stress resistance, and yield as signal molecules. Thus, understanding sugar and organic acid accumulation is of great significance (Chen et al., 2020; Huang et al., 2021; Wen et al., 2022). As mentioned above, the metabolism and accumulation of sugars and organic acids are controlled by complex metabolic pathways and transmembrane transporters involved in multigene responses. Compared with other horticultural crops, such as apple and peach (Li et al., 2012; Zheng et al., 2020), the information on metabolic modulations and accumulation mechanisms related to sugars and organic acids in pineapple is limited. Although some studies have been reported on the changes in key enzymes and relevant biosynthesis genes linked to sugars and organic acids during pineapple fruit development (Saradhuldhat and Paull, 2007; Zhang et al., 2012, 2019; Wu et al., 2022), the metabolic pathways and genes controlling sugar and organic acid accumulation in pineapple fruit remain largely unknown.

The release of the high-quality pineapple genome database paves the way for clarifying the molecular mechanisms in pineapple based on genome-wide transcriptomic analysis (Ming et al., 2015; Wang L. L. et al., 2020). Moreover, with the advances in transcriptomic analysis approaches, a useful method such as weighted gene coexpression network analysis (WGCNA) has made it possible to detect modules of coexpressed genes and key genes responsible for important traits by correlating the transcriptome data with phenotypic data, as reported in watermelon and kiwifruit with different genotypes (Umer et al., 2020; Liao et al., 2021). In addition, although the metabolic phenotype is largely influenced by the environment and difficult to monitor accurately, a rapid and reliable metabolomics approach such as liquid chromatography with tandem mass spectrometry (LC-MS/MS) can detect a range of plant metabolites with high throughput and sensitivity from different biological samples (Hong et al., 2021). Recently, transcriptomics and metabolomics have been integrated to obtain the gene networks and regulatory mechanisms of quality formation in horticultural plants, such as watermelon (Umer et al., 2020) and kiwifruit (Wang et al., 2022). However, little research has been reported on combined RNA sequencing (RNA-Seq) and LC-MS/MS in the study of the molecular mechanisms controlling organic acid and sugar accumulation in pineapple.

In this study, LC-MS/MS and RNA-Seq analyses were used to investigate the primary metabolic dynamics and gene expression profiles of two pineapple cultivars in different fruit developmental stages, focusing on the metabolites and genes associated with sugars and organic acids contributing to fruit quality. Furthermore, WGCNA was performed to construct the regulatory networks and screen related genes controlling the accumulation of sugar and organic acids by the combination of these metabolic data and gene expression profiles. Overall, these data can provide important insights into the regulation of sugar and organic acid metabolism and establish a theoretical basis for flavor improvement in pineapple.

## Results

### Morphological and physiological characteristics of pineapple fruit during development

**FIGURE 1:**
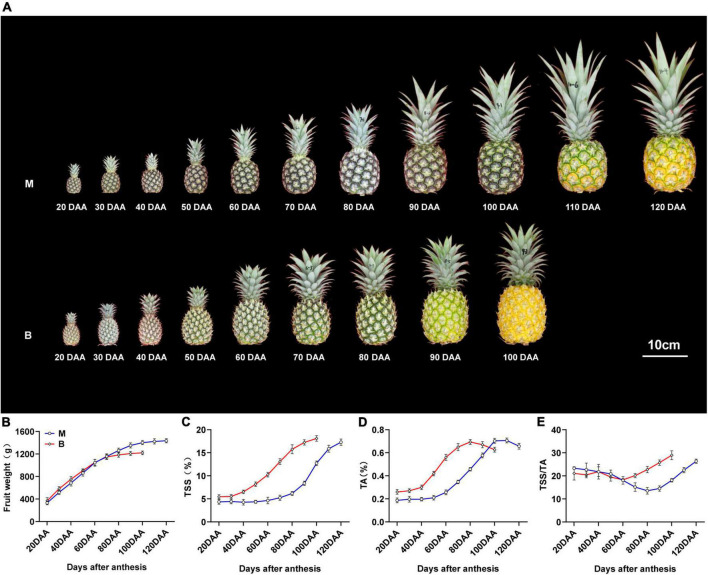
Changes in fruit morphology and physiological index and fruit weight during fruit development and maturity. **(A)** Pineapple cultivars M and B fruits at different developmental stages. **(B)** Fruit weight in the fruit at different developmental stages. **(C)** TSS in the fruit at different developmental stages. **(D)** TA in the fruit at different developmental stages. **(E)** TSS/TA in the fruit at different developmental stages. DAA, days after anthesis; Data were the mean ± standard error from three biological replicate assays.

“Comte de Paris” (B) is an early mature cultivar widely cultivated in China, and “MD-2” (M) is a newly introduced middle mature cultivar. Changes in fruit weight, total soluble solids (TSS), total acids (TA), and TSS/TA ratio during different developmental stages of the two cultivars are shown in Figure 1. The fruit weight of both cultivars consistently increased during the rapid expansion stage [20–90 days after anthesis (DAA) for M, 20–70 DAA for B], and then maintained a limited variation during the mature stage (90–120 DAA for M, 70–100 DAA for B), with a gradual change in fruit color from green to yellow (Figures 1A,B). In common practice, fruit harvest of M is usually at 120 DAA and that of B is at 100 DAA, when more than two-thirds of the fruit has turned yellow and reached 80% maturity.

The total soluble solids (TSS) content of the two varieties increased slightly during the expansion stage, and sharply increased during the maturity stage, and then reached the maximum at harvest time (Figure 1C). The TSS content of M was lower than that of B during the 20–100 DAA, but there was no pronounced difference at harvest time. The total acids (TA) content of both cultivars gradually increased and peaked at 20 days before harvest, and then decreased slightly until harvest (Figure 1D). The TA content of M was higher than that of B during the 20–90 DAA, but there was no significant difference at harvest time. The TSS/TA ratio of M differed slightly from 20 to 80 DAA and then increased till 120 DAA, and the ratio in B differed slightly from 20 to 60 DAA, then increased till 100 DAA (Figure 1E). The differences in TSS, TA and TSS/TA ratio at different stages accounted for the differences in fruit sweet-acidic taste at different stages of the M and B cultivar.

To investigate the soluble sugars and organic acid-associated metabolic modulations throughout the development of pineapple fruit flesh, four developmental stages (40, 80, 100, and 120 DAA; represented by M1, M2, M3, and M4) for M and three developmental stages (40, 80, and 100 DAA, represented by B1, B2, and B3) for B were selected and used for transcriptome sequencing and metabolomic analysis.

### Metabolic profiling of pineapple fruit during development

**FIGURE 2:**
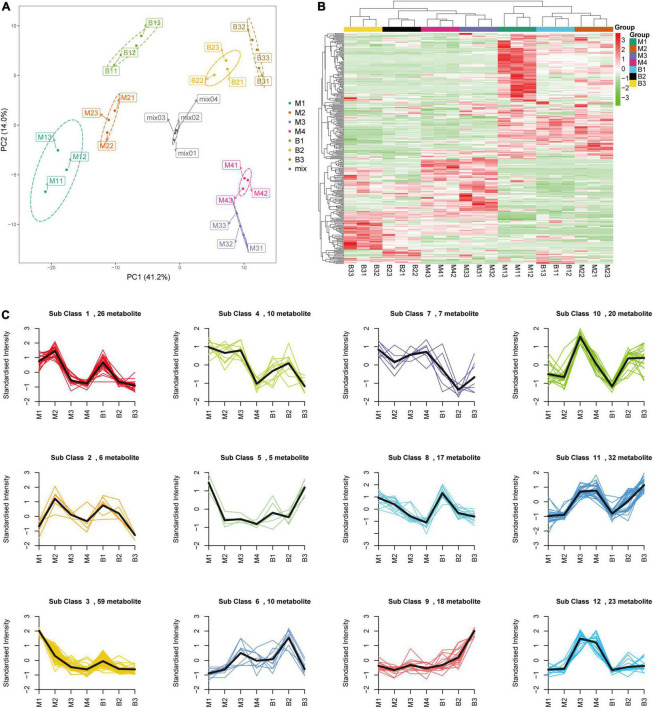
PCA **(A)**, HCA **(B)**, and K-means clustering **(C)** of the metabolites from the developing pineapple flesh. M1, M2, M3, and M4 represented fruit flesh samples for M at 40, 80, 100, and 120 DAA, respectively, B1, B2, and B3 represented fruit flesh samples for B at 40, 80, and 100 DAA, respectively. In the heatmap, each sample was represented by a single column, and each metabolite was visualized in a row. Red showed high abundance, and green exhibited relatively low metabolite abundance. 12 clusters (Sub class 1–12) of 233 differential metabolites were divided based on the dynamic changes of metabolites from different developmental stages for the two cultivars.

Targeted metabolomic analysis based on ultra-performance liquid chromatography-electrospray ionization-tandem mass spectrometry (UPLC-ESI-MS/MS) was performed to investigate the primary metabolite (e.g., sugars and organic acids) dynamics of pineapple fruit during development. A total of 280 primary metabolites, including 56 amino acids and their derivatives, 81 phenolic acids, 31 nucleotides and their derivatives, 24 sugars and alcohols, 28 organic acids, 52 lipids, and 8 vitamins, were identified in our study (Supplementary Table 1). In the principal component analysis (PCA) diagram (Figure 2A), pineapple fruit samples were separated into distinct clusters based on different developmental stages of the two cultivars, indicating the significant differences in metabolite levels among pineapple fruit samples during fruit development. The PCA results showed that the first principal component (PC1) explained 41.16% of the total variance and distinguished samples based on different developmental stages (rapid expansion or mature stages); the second principal component (PC2) separated the two cultivars with a 14.01% variance contribution value. A larger value of PC1 revealed that different developmental stages mainly caused differences in metabolites in samples. In the heatmap (Figure 2B), two main clusters were obtained according to metabolic accumulation during fruit development in the two cultivars. Metabolites such as phenolic acids, amino acids and derivatives and lipids in cluster 1 mainly accumulated at the rapid expansion stages (M1, M2, and B1), while organic acids, saccharides and alcohols, and some phenolic acids in cluster 2 preferentially accumulated at the mature stages (M3, M4, B2, and B3) (Table 1), indicating that metabolite accumulation was stage-specific during the expansion and mature stages of fruit development in pineapple. Moreover, both PCA and hierarchical clustering analysis (HCA) confirmed that the three biological replicates in each group were clustered, indicating the high reliability of the data.

**TABLE 1:** Distribution of 280 primary metabolites identified between cluster 1 and cluster 2 based on HCA.

Clusters	C1	C2	Total
Compounds	216	64	280
Phenolic acids	61	20	81
Lipids	45	7	52
Amino acids and derivatives	49	7	56
Organic acids	17	11	28
Saccharides and alcohols	14	10	24
Nucleotides and derivatives	24	7	31
Vitamin	6	2	8

Differential metabolites were analyzed based on variable importance in projection (VIP) ≥ 1 and | log2 (fold change) | ≥ 1. A total of 233 differential metabolites (18 sugars and alcohols, 24 organic acids, etc.) were identified at different developmental stages of the two cultivars (B1 vs. B2 and B3; B2 vs. B3; M1 vs. M2, M3, M4; M2 vs. M3, M4; M3 vs. M4) (Supplementary Table 2). In the orthogonal partial least squares discriminant analysis (OPLS-DA) results, the Q^2^ values of the nine comparisons were higher than 0.87, indicating the satisfactory predictive capabilities of the models (Supplementary Figures 1A–I). To comprehensively understand the accumulation dynamics of 233 differential metabolites with fruit development in both cultivars, K-means clustering analysis was applied to display 12 distinct subclasses (Figure 2C and Table 2). Among these subclasses, subclasses 5, 7, 9, 10, and 12 accounted for only a small proportion (31%) of metabolites and mainly featured phenolic acids and nucleotides and derivatives, and showed different change trend during the entire period of the fruit development between the two varieties. For subclasses 1, 2, 3, 4, 6, and 8, metabolites were mainly composed of lipids, amino acids and derivatives and phenolic acids, and displayed the significant decreasing during certain periods of the fruit development in both cultivars. However subclass 11 exhibited the significant upregulation of metabolites from the rapid expansion to mature stage in both cultivars, and mainly comprised of phenolic acids, sugars and organic acids, which were associated with the formation of fruit sweet-acidic taste. The change pattern of these metabolites in subclass 11 was similar to that of TSS and TA during the fruit development in both cultivars. Specifically, Suc and citric acid were found to be the predominant sugar and organic acid of many pineapple genotypes, respectively (Lu et al., 2014), and were concentrated in subclass 11. Additionally, chlorogenic acid was considered to be related to antioxidant-associated attributes of pineapple (Arampath and Dekker, 2019), were also grouped in subclass 11.

**TABLE 2:** Distribution of the 233 differentially accumulated metabolites identified among different k-means clusters.

Subclass	S1	S2	S3	S4	S5	S6	S7	S8	S9	S10	S11	S12	Total
Compounds	26	6	59	10	5	10	7	17	18	20	32	23	233
Phenolic acids	7	1	10	3	2	3	3	2	7	1	15	16	70
Lipids	7	2	23	2	1	4	0	0	0	5	0	2	46
Amino acids and derivatives	6	0	13	3	0	0	0	6	2	10	2	3	45
Organic acids	1	3	4	1	0	0	0	3	2	1	7	2	24
Saccharides and alcohols	2	0	4	0	0	0	2	1	1	0	8	0	18
Nucleotides and derivatives	2	0	4	1	2	2	1	4	6	3	0	0	25
Vitamin	1	0	1	0	0	1	1	1	0	0	0	0	5

### Variations among sugars and organic acids in pineapple fruit during development

**FIGURE 3:**
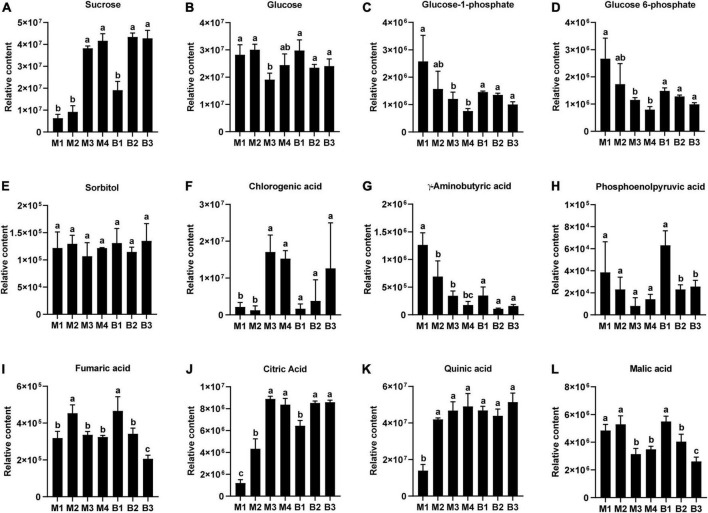
Changes in the relative contents of the main sugars and organic acids in pineapple fruits at four different stages (40, 80, 100, and 120 DAA) for M (M1, M2, M3, and M4) and three different stages (40, 80, and 100 DAA) for B (B1, B2 and B3). **(A)** Sucrose, **(B)** glucose, **(C)** glucose 1-phosphate, **(D)** glucose 6-phosphate, **(E)** sorbitol, **(F)** chlorogenic acid, **(G)** gamma-aminobutyric acid, **(H)** phosphoenolpyruvic acid, **(I)** fumaric acid, **(J)** citric acid, **(K)** quinic acid, **(L)** malic acid. For each cultivar, bars with the same lowercase letter (a, b, or c) indicate no significant differences (*p* < 0.05).

Metabolites likely to be associated with sugar and organic acid metabolism and accumulation in pineapple, included four sugars (Sucrose, glucose 6-phosphate, glucose 1-phosphate, and glucose) an alcohol (sorbitol), six organic acids (citric acid, quinic acid, malic acid, fumaric acid, gamma-aminobutyric acid, and phosphoenolpyruvic acid), and a phenolic acid (chlorogenic acid) (Figure 3). For the four sugars and alcohol, Suc content was low during the rapid expansion stages and increased significantly during the mature stages in the two cultivars (Figure 3A), whereas Glc content was high during the rapid expansion stages but displayed a slight decreasing trend during the mature stages in the two cultivars (Figure 3B). Developmental changes in Glc-1-phosphate and Glc 6-phosphate content showed a decrease, whereas sorbitol content displayed no significant changes in the two cultivars (Figures 3C–E). Among the six organic acids, increasing trends were observed in the content of citric acid and quinic acid during fruit development in the two cultivars, and the citric acid level dramatically increased from the expansion to mature stages for both cultivars (Figures 3J,K). In contrast, the levels of malic acid, fumaric acid, GABA, and phosphoenolpyruvic acid displayed decreasing trends as the fruits moved toward maturity in the two cultivars, and malic acid levels exhibited significant downregulation from the expansion to mature stages for the two cultivars (Figures 3G–I,L). In addition, an increasing trend in the chlorogenic acid level was observed during fruit development in the two cultivars, and the chlorogenic acid content significantly increased from the expansion to mature stages in M cultivar (Figure 3F). Consistent with the previous results, Suc and citric acid contents were the highest in mature fruit of the soluble sugars and organic acids in both cultivars, respectively (Table 3) (Lu et al., 2014). Altogether, the changes in the Suc and citric acid contents during fruit development in both cultivars were consistent with that of the TA and TSS contents, respectively, indicating that Suc and citric acid could be crucial for the formation of fruit sweet-acidic taste of both cultivars.

**TABLE 3:** Content of sugars and organic acids at different developmental stages in M and B cultivar (mg⋅g^–1^ FW) and the values shown are the mean ± SD.

	M1	M2	M3	M4	B1	B2	B3
Sucrose	14.22 ± 0.58	20.38 ± 0.55	68.9 ± 0.52	74.06 ± 0.66	25.92 ± 0.68	58.03 ± 0.98	63.23 ± 0.52
Fructose	5.41 ± 0.26	6.62 ± 0.24	11.51 ± 0.71	13.29 ± 0.47	12.61 ± 0.27	22.22 ± 0.44	28.14 ± 0.35
Glucose	6.67 ± 0.30	7.71 ± 0.31	14.02 ± 0.28	15.81 ± 0.50	14.44 ± 0.68	24.74 ± 0.40	31.50 ± 0.83
Citric acid	1.13 ± 0.16	2.29 ± 0.16	3.78 ± 0.15	3.35 ± 0.27	1.53 ± 0.12	3.37 ± 0.20	2.77 ± 0.32
Quinic acid	0.24 ± 0.02	0.67 ± 0.03	0.83 ± 0.02	0.93 ± 0.06	0.47 ± 0.03	0.65 ± 0.02	0.78 ± 0.02
Malic acid	1.57 ± 0.05	1.69 ± 0.03	1.15 ± 0.02	1.04 ± 0.04	1.64 ± 0.04	1.12 ± 0.02	0.85 ± 0.02

### Transcriptome profiling of pineapple fruit during development

**FIGURE 4:**
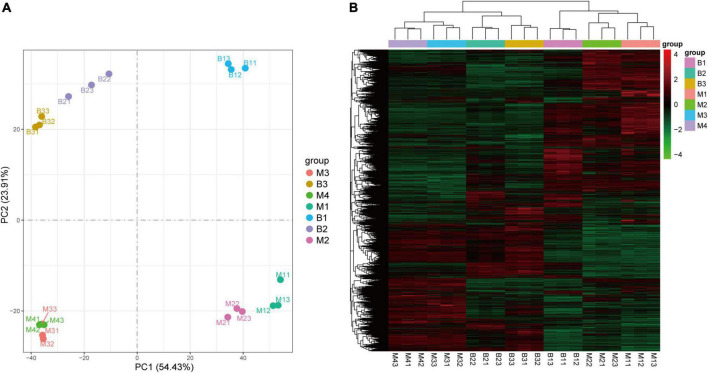
PCA **(A)** and HCA **(B)** of genes in pineapple fruits at different developmental stages of M and B. M1, M2, M3, and M4 represented fruit flesh samples for M at 40, 80, 100, and 120 DAA, respectively, B1, B2, and B3 represented fruit flesh samples for B at 40, 80, and 100 DAA, respectively.

To investigate the molecular basis underlying pineapple quality-associated metabolism changes, 21 cDNA libraries for different developmental stages of the two cultivars were constructed by RNA-Seq. The details of RNA-Seq data for each sample are shown in Supplementary Table 3. After filtering low-quality reads in each library, 7.48 Gb clean reads on average for each individual sample lines were generated with >89% Q30 base percentage. When clean reads were mapped to the pineapple genome, >80% of clean reads were matched (Supplementary Table 3). In addition, both PCA and HCA of the transcriptome confirmed a clear separation between the groups and a good correlation within the groups (Figures 4A,B), revealing the reliability of the transcriptome data.

### Identification of coexpressed gene networks by weighted gene coexpression network analysis

**FIGURE 5:**
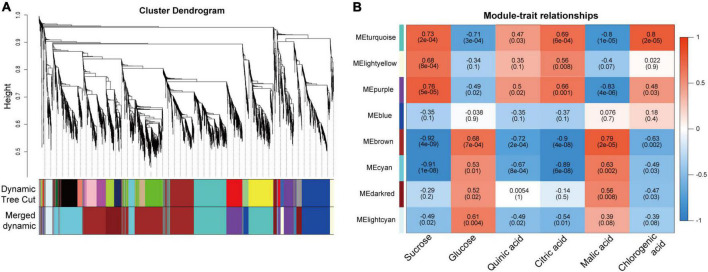
Result of gene coexpression modules associated with sugars and organic acids in pineapple fruits at different developmental stages of M and B. **(A)** Clustering dendrogram presenting 11 modules of coexpressed genes based on WGCNA. **(B)** Correlations coefficient and significance between modules and sucrose, glucose, quinic acid, citric acid, malic acid, chlorogenic acid, where each grid contained the corresponding correlation and *p*-value.

To understand the regulatory mechanisms of soluble sugars and organic acids during pineapple fruit developmental stages, WGCNA was used to identify the coexpressed gene networks. After removing low- expressed genes from all identified genes (Supplementary Table 4), 3,723 genes with FPKM values were entered into the WGCNA module. Here, Suc, Glc, quinic acid, citric acid, malic acid, and chlorogenic acid content at different stages of the two cultivars were taken as phenotypic data for the analysis of gene module–trait correlations. A total of 8 distinct modules marked with different colors were produced according to the similar coexpressed patterns of each gene (Figure 5A and Supplementary Table 5). Detailed information of the gene module–trait correlations is shown in Figure 5B. Interestingly, the turquoise and purple modules showed significant positive correlations with Suc, citric acid, and chlorogenic acid, which preferentially accumulated at mature stages (B2, B3, M3, and M4), whereas the brown and cyan modules presented significant negative correlations with these metabolites mainly accumulating at the mature stages. In contrast, the turquoise and purple modules showed negative correlations with Glc and malic acid, which mainly accumulated at the rapid expansion stages (B1, M1, and M2), whereas the brown and cyan modules showed significant positive correlations with those metabolites mainly accumulating at the rapid expansion stages. Thus, the genes in the four modules might play a vital role in modulating soluble sugar and organic acid metabolism in the pineapple fruit developmental stages.

### Quantitative real-time PCR verification of gene expression

**FIGURE 6:**
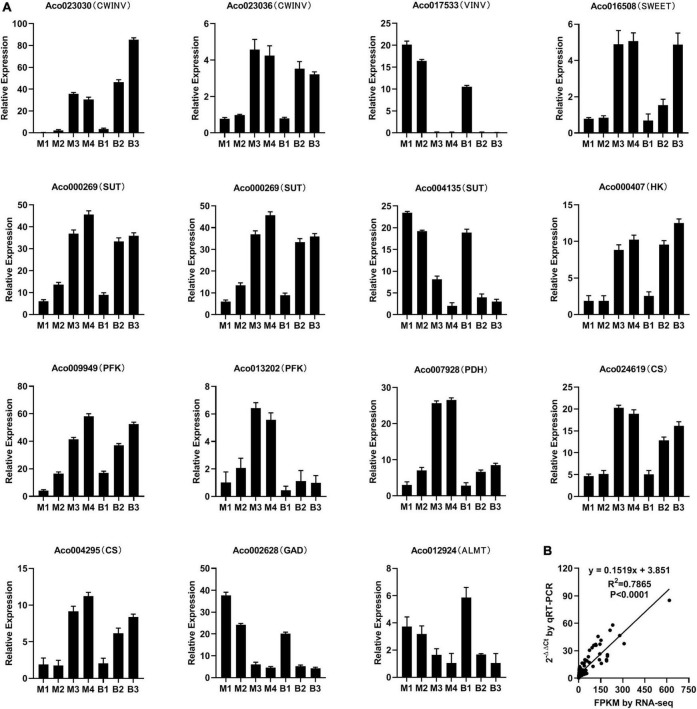
Relative expression of 15 genes involved in sugar and organic acid metabolism and transport during different developmental stages of M and B by qRT-PCR **(A)** and correlation analysis between RNA-Seq and qRT-PCR data **(B)**.

To verify the RNA-Seq data, 15 genes linked to sugar and organic acid accumulation in pineapple flesh were subjected to qRT-PCR analysis (Figure 6). qRT-PCR demonstrated that the expression trends of the genes were consistent with the RNA-Seq analysis results (Figure 6A). Correlation analysis (*R*^2^ = 0.79, *P* < 0.0001) between RNA-Seq and qRT-PCR data indicated the reliability of the RNA-Seq data (Figure 6B).

### Genes related to sugar metabolism and accumulation in pineapple fruit

**FIGURE 7:**
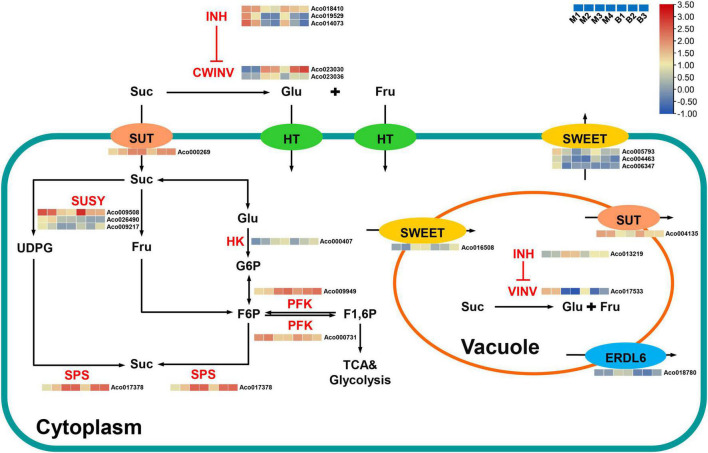
Regulatory model and expression levels of the genes related to sucrose metabolism and accumulation in pineapple fruits. From blue to red in the heatmap indicates the expression levels of the genes ranging from low to high in two pineapple cultivars throughout fruit development. M1, M2, M3, and M4 represented fruit flesh samples for M at 40, 80, 100, and 120 DAA, respectively, and B1, B2, and B3 represented fruit flesh samples for B at 40, 80, and 100 DAA, respectively. CWINV, cell wall invertase; CWINH, cell-wall invertase inhibitor; VINV, vacuolar acid invertase; VINH, vacuolar acid invertase inhibitor; SUSY, sucrose synthase; SPS, sucrose phosphate synthase; HK, hexokinase; PFK, phosphofructokinase; SUT, sucrose transporters; SWEET, sugars will eventually be exported transporter; ERDL6, glucose exporter early response to dehydration like 6; Suc, sucrose; Glc, glucose; Fru, fructose; UDPG, UDP-glucose; G6P, glucose-6-phosphate; F6P, fructose-6-phosphate; F1, 6P, fructose-1, 6-phosphate.

Suc, the main contributor to pineapple sweetness (Zhang et al., 2012, 2019), was the predominant soluble sugar of mature fruits in both cultivars. Interestingly, Suc accumulation was strongly negatively associated with the brown (*r*^2^ = –0.92) and cyan (*r*^2^ = –0.91) modules and highly positively correlated with the turquoise (*r*^2^ = 0.73) and purple (*r*^2^ = 0.76) modules. Finally, 21 candidate genes linked to sugar metabolism and sugar transport in pineapple (Supplementary Table 6 and Figure 7). Acid invertases (INVs), including cell wall invertase (CWINV) and vacuolar acid invertase (VINV), participate in the irreversible hydrolysis of Suc in the apoplast or vacuole. For Suc hydrolysis, two CWINV genes (*Aco023030*, *Aco023036*) in the turquoise and purple modules and a VINV gene (*Aco017533*) from the cyan module in pineapple were first identified. The expression levels of the two CWINV genes were positively correlated with Suc content, whereas the VINV gene expression level was strongly negatively correlated with Suc content during the developmental stages. Invertase inhibitor (INH), an inhibitory protein binding to the active site (Suc binding site) of INV, includes cell wall INHs (CWINHs) and vacuolar INHs (VINHs) based on their subcellular location (Wang et al., 2020; Kawaguchi et al., 2021). Here, of the four INH genes identified, the expression levels of *Aco018410* from the cyan module and *Aco019529* and *Aco014073* from the brown module were strongly negatively correlated with Suc content, and the *Aco013219* expression level from the turquoise module was positively correlated with Suc content during fruit development.

Within the Suc–Suc cycle, three Suc synthase (SUSY) genes (*Aco009508*, *Aco026490*, *Aco009217*) in the brown and cyan modules were strongly negatively correlated with Suc content during fruit development. In addition, one Suc phosphate synthase (SPS) gene (*Aco017533*) and one hexokinase (HK) gene (*Aco000407*) were found in the turquoise module, and their expression levels were positively correlated with Suc content during fruit development. Of the two phosphofructokinase (PFK) genes identified, *Aco000731* in the cyan module was strongly negatively correlated with Suc content, whereas *Aco009949* was positively correlated with Suc content throughout fruit development. In addition, seven sugar transporter genes, including two sucrose transporter (SUT) genes, four sugars will eventually be exported transporter (SWEET) genes, and one the Glc exporter early response to dehydration like 6 (ERDL6) gene, were examined. The expression level of one SUT gene (*Aco000269*) from the turquoise module was positively correlated with Suc content, whereas that of the other SUT gene (*Aco004135*) from the brown module was negatively correlated with Suc content during fruit development. Among the four SWEET genes, three SWEET genes (*Aco005793*, *Aco004463*, *Aco006347*) that negatively correlated with Suc were present in the brown module, and one SWEET gene (*Aco016508*) that positively correlated with Suc was found in the turquoise module. An ERDL6 gene (*Aco018780*) that positively correlated with Suc was found in the turquoise module.

### Genes correlated with organic acid metabolism and accumulation in pineapple fruit

**FIGURE 8:**
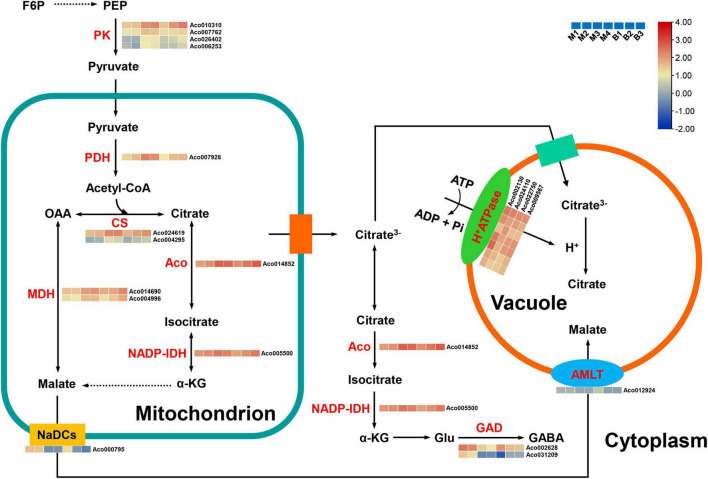
Regulatory model and expression levels of the genes related to citric acid and malic acid metabolism and accumulation in pineapple fruits. From blue to red in the heatmap indicates the expression levels of the genes ranging from low to high in two pineapple cultivars throughout fruit development. M1, M2, M3, and M4 represented fruit flesh samples for M at 40, 80, 100, and 120 DAA, respectively, and B1, B2, and B3 represented fruit flesh samples for B at 40, 80, and 100 DAA, respectively. PK, pyruvate kinase; PDH, pyruvate dehydrogenase; CS, citrate synthase, ACO, aconitase; MDH, malate dehydrogenase; NADP-IDH, isocitrate dehydrogenase NADP; GAD, glutamate decarboxylase; ALMT, aluminum-activated malate transporter; NaDCs, sodium-dependent dicarboxylate transporter. PEP, phosphoenolpyruvate; a-KG, a-ketoglutarate; Glu, glutamate; GABA, γ-aminobutyric acid.

Citric acid, contributing to the sour taste of pineapple (Saradhuldhat and Paull, 2007; Lu et al., 2014), was also the major organic acid of mature fruits in the two cultivars. Altogether, 19 candidate genes were first identified as being related to organic acid metabolism and transport in pineapple (Supplementary Table 7 and Figure 8). For citrate synthesis, nine genes, including four pyruvate kinase (PK) genes (*Aco010310*, *Aco007762*, *Aco026402*, *Aco006253*), one pyruvate dehydrogenase (PDH) gene (*Aco007928*), two citrate synthase (CS) genes (*Aco024619*, *Aco004295*), one aconitase (ACO) gene (*Aco014852*), and one isocitrate dehydrogenase NADP (IDH-NADP) gene (*Aco005500*), were identified. These nine genes were present in the turquoise or purple module, and their expression levels were highly positively correlated with citrate content (*r*^2^ = 0.73 or *r*^2^ = 0.76). For citrate degradation, 16 glutamate decarboxylase (GAD) genes were present in the transcriptome data, but only two GAD genes *Aco002628* and *Aco031209* were present in the brown and cyan modules, respectively, and were strongly negatively associated with citrate content (*r*^2^ = –0.90, *r*^2^ = –0.89). For citrate transport, four genes from the turquoise or purple module were identified as H^+^-ATPase genes (*Aco002130*, *Aco024110*, *Aco022750*, *Aco009567*) involved in the diffusion of citrate into the vacuole. The expression levels of these four genes displayed positive correlations (*r*^2^ = 0.73 or *r*^2^ = 0.76) with citrate content. For malate metabolism and transport, two malate dehydrogenase (MDH) genes (*Aco014690*, *Aco004996*) in the purple module had a highly negative correlation (*r*^2^ = –0.83) with malate content, whereas one aluminum-ALMT gene (*Aco012924*) and one sodium-dependent dicarboxylate transporter (NaDC) gene (*Aco000795*) in the brown module were highly positively correlated (*r*^2^ = 0.79) with malate content.

## Discussion

The metabolism of sugars and organic acids is essential for fruit development, and their accumulation in fleshy fruits is central to the taste and quality of fruits (Chen et al., 2020; Huang et al., 2021; Wen et al., 2022). Primary metabolites, such as Suc, citrate, and malate, are important metabolites that not only provide energy and substrates for respiratory processes but are also a major source of fleshy fruit sweetness and acidity (Igamberdiev and Eprintsev, 2016; Wen et al., 2022). However, the description of the pineapple fruit metabolic spectrum mainly focuses on the mature stage or volatile metabolites (Steingass et al., 2015; Hong et al., 2021), and the global metabolic spectrum of different fruit developmental stages based on UPLC-ESI-MS/MS has not been identified. In this study, 280 primary metabolites were detected and annotated, and 233 differential metabolites were found in the two cultivars at different developmental stages. Metabolite accumulation in pineapple fruit was stage-specific (expansion and mature stages), which is consistent with the result in kiwifruit (Wang et al., 2022). Furthermore, by comprehensive transcriptomic and metabolomic analyses during fruit development, key metabolic pathways and complex genetic factors regulating sugar and organic acid accumulation were identified. In addition, the generated dataset could identify other pathways and regulatory genes related to flavor metabolism. Altogether, our finding will not only enhance the understanding of the complex regulatory mechanisms of sweet-acidic taste formation in pineapple but also provide valuable reference for the high-quality genetic improvement of pineapple.

### Reasons for sucrose accumulation during pineapple fruit development

In this study, Suc was the predominant sugar in mature fruits and presented more remarkable changes during fruit development compared with the other sugars contributing to sweetness such as hexose, which was similar to previous studies (Zhang et al., 2012, 2019). As in apple and kiwifruit (Li et al., 2012; Vimolmangkang et al., 2016), the dataset in this study distinctly showed that Suc metabolism was highly developmentally modulated in pineapple. At the fruit expansion stage of the two cultivars, high expression levels of six genes on sugar metabolism were found, including three SUSY genes and one PFK genes (*Aco000731*), which may result in rapid metabolism of the imported Suc to supply the energy and intermediates for expansion and growth (Li et al., 2012), and one VINV gene, which could hydrolyze unmetabolized Suc to hexose for vacuolar accumulation. Thus, Suc content was low. As the pineapple fruit transitions to the mature stage, the expression levels of these six genes notably decreased, whereas the expression levels of two CWINV genes, one SPS gene, one HK gene, one PFK genes (*Aco009949*), one SWEET gene (*Aco016508*), one SUT gene (*Aco000269*), and one ERDL6 gene significantly increased. This process might be involved in CWINV-mediated Suc apoplast hydrolysis, SPS-mediated Suc cytoplasmic resynthesis, and SUT, SWEET, and ERDL6-mediated sugar transport. Thus, Suc content was high. Collectively, Suc accumulation in pineapple fruit is the result of coordinated actions of multiple genes.

CWINV catalyze the irreversible hydrolysis of Suc into glucose and fructose in the apoplast, determining the ratio of Suc: (glucose + fructose) in the apoplast of fruits during ripening (Walker et al., 2021). High CWINV activity contributed to the maintenance of a high intracellular-to-extracellular Suc ratio and the production of a negative water potential, leading to the continuous export of Suc into the apoplast (Minami et al., 2021). Apoplastic hexose could be directly transported into the cytosol to participate in Suc resynthesis (Miron et al., 2002). In this study, we identified two CWINV genes, which were highly expressed in fruits during mature stages, and displayed similar expression patterns to that of the Suc during fruit development, indicating that Suc hydrolysis in the apoplast mediated by CWINVs might be a critical step for Suc accumulation in pineapple, as reported in strawberry fruit (Yuan et al., 2021). CWIN activity could be regulated by invertase inhibitors (INHs) at the posttranslational level (Ru et al., 2020). Recently, the knockout of *SlCWINH1* by CRISPR/Cas9 significantly increased the contents of Suc and hexose in genome-edited tomato line 193-3, possibly caused by higher CWINV activity in the apoplast of tomato fruits (Kawaguchi et al., 2021). In the present study, significant negative correlations of three INH genes with Suc implied that they might act as essential regulators of apoplastic Suc hydrolysis to control Suc accumulation in pineapple fruit. Unlike CWINVs, VINV mainly catalyze the irreversible hydrolysis of Suc into glucose and fructose and helps to maintain cell osmotic potential in vacuole (Ruan et al., 2010). The presence of VINV could contribute to increasing turgor pressure of expanding cells in fruits before ripening (Walker et al., 2021). The negative correlation of the VINV gene (*Aco017533*) with Suc content in this study showed that Suc was hydrolyzed into hexose by VINV in the vacuole at the expansion stages with less accumulation of Suc, and less likely to be hydrolyzed into hexose by VINV in the vacuole at the mature stages with Suc accumulation. Studies also show that post-translational limitation of VIN by INH contributes to control Suc content in fruits such as peach (Wang et al., 2020) and pear (Ma et al., 2020). Interestingly, the positive correlation of the INH gene (*Aco013219*) with Suc content of pineapple fruit suggested an interaction between Aco013219 and VINV, thereby participating in regulating pineapple fruit development and Suc accumulation. Hence, further studies are necessary to explore the interaction between INVs and INHs and their function in pineapple sugar accumulation.

Sugar transporters, the main members of the channels regulating sugar influx or efflux, significantly affect fruit sugar accumulation (Vimolmangkang et al., 2016; Aslam et al., 2019; Wen et al., 2022). Downregulation of *PpSUT4* expression in mature fruits showed the vacuolar efflux of Suc in peach (Zanon et al., 2015a,b). The *MdSUT4.1* expression level was negatively correlated with fruit sugar content, and its overexpression in strawberry and apple callus reduced sugar levels, suggesting that it was involved in remobilizing vacuolar sugar (Peng et al., 2020). In melon fruit, the high expression of the *CmSUT3* gene may be involved in the unloading of Suc in the apoplast (Wen et al., 2022). In the present study, the SUT gene (*Aco000269*) expression level showing a positive correlation with Suc content suggested that it was involved in apoplast unloading of Suc, whereas the SUT gene (*Aco004135*) expression level displaying a negative correlation with Suc content suggested that it was involved in Suc efflux from the vacuole membrane to the cytoplasm. Furthermore, the positive correlation between the SWEET gene (*Aco016508*) expression level and Suc content in this study showed that it might mediate Suc release into the apoplast and contribute to Suc accumulation in pineapple fruit based on a study in *Citrus* (Feng et al., 2021). The expression levels of the other three SWEET genes were negatively correlated with Suc content, which was consistent with previous studies (Guo et al., 2018). Thus, these three SWEET genes might transport Suc across the plasma membrane and be involved in Suc unloading as described in white pear (Li et al., 2017) or tomato (Zhang et al., 2021). In addition, although the hexose transporter MST1 gene has been identified in pineapple, its expression level at different fruit developmental stages remains unknown (Antony et al., 2008). ERDL6 is mainly responsible for transferring Glc from the vacuole to the cytosol. In apple, the expression levels of multiple *MdERDL6* family members were strongly positively related to Suc and Fru concentrations, and MdERDL6 mediated the Glc efflux to the cytoplasm, resulting in the accumulation of Suc and Fru in vacuolar by upregulating *tonoplast sugar transporter* expression (Zhu et al., 2021). Here, an *ERDL6* similar to *MST1* was identified, and its expression was positively correlated with Suc content during fruit development. This result further demonstrates that ERDL6 could be involved in regulating Suc accumulation in pineapple fruit. However, other hexose transporters, such as tonoplast sugar transporters and vacuolar Glc transporter, which are significantly related to sugar content of other fruits (Zhu et al., 2021; Wen et al., 2022), were not identified in the key modules. Nevertheless, we noticed that expression pattern of *ERDL6* (*Aco018780*) was similar to that of the *SWEET* (*Aco016508*), and SWEETs are regarded as having the characteristics of Suc transportation in the vacuolar membrane (Wen et al., 2022). It was speculated that, ERDL6-mediated Glc transportation could enhance the Suc accumulation in the vacuole of pineapple fruit mediated by the coordination of *ERDL6* (*Aco018780*) and *SWEET* (*Aco016508*).

### Reasons for organic acid accumulation during pineapple fruit development

In the present study, early accumulation of malate was observed during the rapid expansion stages, and citrate content notably increased, whereas malate content significantly decreased as the fruits matured. In total, citric acid content were the highest in mature fruit of the organic acids in both cultivars. Thus, the citrate levels were also responsible for fruit acidity in both cultivars, which was consistent with previous research on pineapple (Lu et al., 2014; Wen et al., 2022). Owing to the vital role of organic acids in the regulation of osmotic pressure, stress resistance, and fruit quality (Huang et al., 2021), identifying the key genes and metabolic pathways regulating organic acid accumulation in pineapple is essential.

Citrate accumulation in fruit is controlled by citrate synthesis (Umer et al., 2020). In the last step of glycolysis, PK catalyzes the conversion of phosphoenolpyruvate (PEP) to pyruvate, and much pyruvate in the cytosol is transported to the mitochondria; subsequently, the PDH complex (composed of PDH, dihydrolipoyl transacetylase, and dihydrolipoyl dehydrogenase) converts pyruvate to acetyl-CoA, which determines the CS reaction rate in the TCA cycle (McCommis and Finck, 2015; Zheng et al., 2020). In peach fruit, the downregulation of PDH kinase gene (*PDK*) expression inhibited PDH and the upregulation of *PK* expression in high-acid cultivars promoted citrate accumulation by the pyruvate-to-acetyl-CoA-to-citrate pathway (Zheng et al., 2020). A previous study in pineapple fruit revealed that the changes in CS and ACO activities coincided with citrate content during fruit development, and the decrease in CS activity and the increase in ACO activity were responsible for the reduction in the acidity in the low-acid clone compared with the high-acid clone (Saradhuldhat and Paull, 2007). In this study, four PK genes, one PDH gene, two CS genes, one Aco gene, and one IDH-NADP gene were positively correlated with citrate content. Thus, these nine genes are likely to play an important role in citrate synthesis during pineapple fruit development by the pyruvate-to-acetyl-CoA-to-citrate pathway.

Citrate degradation by the GABA pathway in the cytosol is also a key player in fruit acidity (Batista-Silva et al., 2018). GADs catalyze the decarboxylation of glutamate to GABA and are related to citrate accumulation, and the higher expression of GAD genes contribute to the decrease in fruit acidity at the late stage of development (Liu et al., 2014; Feng et al., 2021). Low-acid cultivars exhibited citrate degradation attributed to the upregulation of GAD gene expression in peach (Zheng et al., 2020). In this study, two GAD genes were notably negatively correlated with citrate content, suggesting that these two genes are crucial for citrate degradation by the GABA pathway during pineapple fruit development. Moreover, tonoplast proton pumps such as vacuolar-type and p-type ATPases play major roles in driving the facilitated diffusion of citrate into the vacuole (Huang et al., 2021). For instance, *CitPH1* and *CitPH5* encoding P-ATPase were expressed in acid varieties, whereas their expression was significantly reduced in acidless varieties, regulating hyper-acidification in citrus fruits (Strazzer et al., 2019). Another *CsPH8* gene in citrus has also been identified to play a role in determining citrate content or acidity (Shi et al., 2015, 2019). In the present study, one P-type ATPase gene and three H^+^-ATPase genes were identified, and their expression levels were positively related with citrate content, suggesting that they might play central roles in citrate accumulation by driving citrate into the vacuole in pineapple. Besides, malate-related genes were identified in this study. Here, two MDH genes were positively correlated with malate content, whereas two malate-related transporter genes, including *NaDC* and *ALMT*, were significantly negatively correlated with malate content. This result showed that degradation and transport might regulate the malate content of pineapple.

## Materials and methods

### Plant materials

The conventional dominant cultivar “Comte de Paris” (B) belonging to “Queen,” and excellent hybrid cultivar “MD-2” (M) belonging to “Smooth Cayenne,” were used as experimental materials, which planted in the pineapple resource bank of South Subtropical Crops Research Institute (E 110°16′, N 21°10′, Zhanjiang, China) in 2019. All pineapple plants were uniformly managed during fertilization, irrigation, and disease and pest control. At the full florescence period, 150 pineapple plants per cultivar with normal growth and consistent flowering period were selected and tagged. The fruits per cultivar were randomly sampled at 10 a.m. every 10 days until harvest from 20 DAA. M at 11 developmental stages (20, 30, 40, 50, 60, 70, 80, 90, 100, 110, 120 DAA) and B at nine developmental stages (20, 30, 40, 50, 60, 70, 80, 90, 100 DAA) were collected (Figure 1A). The fruit weight, TSS and TA were determined throughout fruit development. Flesh from M at four developmental stages (40, 80, 100, 120 DAA) and B at three developmental stages (40, 80, 100 DAA) were collected according to the methods described in a previous study (Zhang et al., 2012) and immediately frozen in liquid nitrogen and stored at –80°C for further metabolic, transcriptomic, and qRT-PCR analyses. Measurements were made at each developmental stage with three biological replicates and nine fruits in each replicate.

### Total soluble solids, total acids, sugars and organic acids measurement

The indicators of fruit quality mainly include total soluble solids (TSS), total acids (TA), TSS/TA ratio, sugars (glucose, fructose and sucrose) and organic acids (malic, citric and quinic acid). TSS (Brix%) was determined by using a hand-held refractometer (ATC-20E, Atago, Tokyo, Japan). Contents of TA, sugars and organic acids were determined by referring to our previous methods reported (Lu et al., 2014).

### Targeted metabolomics analysis

The extraction, determination, and analysis of metabolites in the flesh samples were performed as previously described (Chen et al., 2013; Zou et al., 2020; Hong et al., 2021). In brief, the frozen fruit flesh samples were weighed, smashed, and extracted in 70% methanol solution for overnight at 4°C. Then, flesh extracts were centrifuged and filtered, and the resulting flesh samples were assessed by UPLC-ESI-MS/MS. During analysis, each of the 10 detected samples included a quality control sample using mixed flesh extracts to monitor repeatability and stability.

All primary metabolites were identified based on MetWare database^1^ and quantified by multiple reaction monitoring. PCA, HCA, and OPLS-DA were performed in R to study differences and reliability of metabolites from the 21 flesh samples. VIP in the OPLS-DA model was used to identify the differential metabolites (VIP ≥ 1 and | log2 (fold change) | ≥ 1). After standardized and centralized treatment, K-means analysis of differential metabolites was performed to study the relative content changes of metabolites in different samples.

### Transcriptomics analysis

Total RNA was extracted from 21 frozen samples, and 21 cDNA libraries were constructed and sequenced on the Illumina Hiseq2000 platform with the generation of 150 bp paired-end reads as previously described (Umer et al., 2020). After processing the original and low-quality data (raw reads), clean reads were acquired and aligned to the pineapple reference genome^2^ (Xu et al., 2018). The transcripts were annotated according to this pineapple genome database or the STRING database. The gene expression level was determined using the FPKM values based on gene lengths and read counts that were mapped to genes. In addition, both PCA and HCA were performed to evaluate differences between groups and the repetition of samples within groups.

### Weighted gene coexpression network analysis

Coexpression network modules were constructed using the WGCNA package in R (Langfelder and Horvath, 2008; Umer et al., 2020). After introducing all genes into the WGCNA package, 3,723 genes with coefficient of variation (CV) > 0.5 and CV < standard deviation (sd) were screened to obtain the coexpression modules with the power of 6, minModuleSize of 30, and mergeCutHeight of 0.25 (Dai et al., 2021). After calculating the eigengene value of each module, the metabolic phenotype data was introduced, and the associations between phenotypes and gene modules during fruit development were obtained.

### Verification of next-generation RNA sequencing data

The expression levels of the genes associated with sugar and organic acid accumulation were measured by qRT-PCR on a LightCycler480 II System (Roche, Switzerland) using primers listed in Supplementary Table 8. After the isolation of RNA of the 21 pineapple flesh samples, cDNA lines were generated using a BioTeke (Beijing, China) SupermoIII RT kit. qRT-PCR was performed with a DyNAmo Flash SYBR Green qPCR kit (Thermo Fisher Scientific, Waltham, MA, United States), following a previously described protocol (Zhang et al., 2019; Wu et al., 2022). Actin (GenBank number HQ148720) was used as a reference gene (Zhang et al., 2019), and the 2^–ΔΔCT^ method was used to determine the gene expression levels.

## Conclusion

A comprehensive analysis of the metabolome and transcriptome of two pineapple cultivars during fruit development was performed to explore the dynamic changes in taste-associated metabolites and the molecular mechanisms controlling sweet-acidic taste during pineapple fruit development based on WGCNA. Suc and citrate were the predominant components of sugars and organic acids in mature fruits of both cultivars. At least 15 candidate genes related to sugar metabolism and accumulation were first identified in pineapple, and their expression levels were significantly related to Suc accumulation, including four *INHs* and three *INVs* related to irreversible hydrolysis of Suc, one *HK* and two *PFKs* participating in the Suc–Suc cycle in the cytoplasm, and two *SUTs* and one *SWEET* associated with sugar transporters. As for the metabolism and accumulation of organic acids, 19 candidate genes were first identified in pineapple, and their expression levels were significantly correlated with citric acid accumulation, including four *PKs*, one *PDH*, two *CSs*, one *ACO* and one *NADP-IDH* related to citrate synthesis, two *GADs* related to citrate degradation, two *MDHs* related to malate metabolism, and four H^+^-ATPase*s*, one *ALMT* and one *NaDC* associated with acid transport. The key metabolic pathways and gene network for sugar and organic acid accumulation in developing pineapple fruit are shown in Figures 7, 8.

## Data availability statement

The original contributions presented in this study are publicly available. This data can be found here: NCBI, PRJNA851558.

## Author contributions

XZ and AG supervised the research. YG, YY, and XC prepared the samples and performed the experiments. YG, JW, and QW analyzed the data, wrote, and revised the manuscript. SL provided the materials. All authors contributed to the article and approved the submitted version.

## Footnotes

1
www.metware.cn

2
http://pineapple.angiosperms.org/pineapple/html/index.html

## Supplementary material

Supplementary Figure 1 OPLS-DA model plots and loading plots for the M1 vs. M2 **(A)**, M1 vs. M3 **(B)**, M1 vs. M4 **(C)**, M2 vs. M3 **(D)**, M2 vs. M4 **(E)**, M3 vs. M4 **(F)**, B1 vs. B2 **(G)**, B1 vs. B3 **(H)**, B2 vs. B3 **(I)** during fruit development of “MD-2” and “Comte de Paris”.

## Abbreviations

Suc: sucrose
Glc: glucose
Fru: fructose
PEP: phosphoenolpyruvate
GABA: gamma-aminobutyric acid
SUT: sucrose transporter
INV: acid invertase
CWINV: cell wall invertase
INH: invertase inhibitor
SUSY: sucrose synthase
SPS: sucrose phosphate synthase
HK: hexokinase
PFK: phosphofructokinase
VINV: vacuolar acid invertase
MST: monosaccharide transporter-like
SWEET: sugars will eventually be exported transporter
ERDL6: the glucose exporter early response to dehydration like 6
CS: citrate synthase
ACO: aconitase
MDH: malate dehydrogenase
ICDH: isocitrate dehydrogenase
ALMTs: aluminum-activated malate transporters
PFK: phosphofructokinase
PDH: pyruvate dehydrogenase
PK: pyruvate kinase
NADP-IDH: isocitrate dehydrogenase NADP
GAD: glutamate decarboxylase
H^+^-ATPase: vacuolar-type and p-type ATPase
NaDCs: sodium-dependent dicarboxylate transporter
RNA-Seq: next-generation RNA sequencing
LC-MS/MS: metabolomics
PCA: principal component analysis
HCA: hierarchical cluster analysis
VIP: variable importance in projection
MRM: multiple reaction monitoring
WGCNA: weighted gene coexpression network analysis
OPLS-DA: orthogonal partial least squares discriminant analysis
QC: quality control
qRT-PCR: quantitative real-time PCR
FPKM: fragments per kilobase of transcript per million fragments mapped.
