# Supplementary figures and images for: Metabolomic and transcriptomic analyses reveal the mechanism of sweet-acidic taste formation during pineapple fruit development
Source: Front Plant Sci. 2022 Sep 8;13:971506. doi: 10.3389/fpls.2022.971506 (PMC9493369; doi:10.3389/fpls.2022.971506)

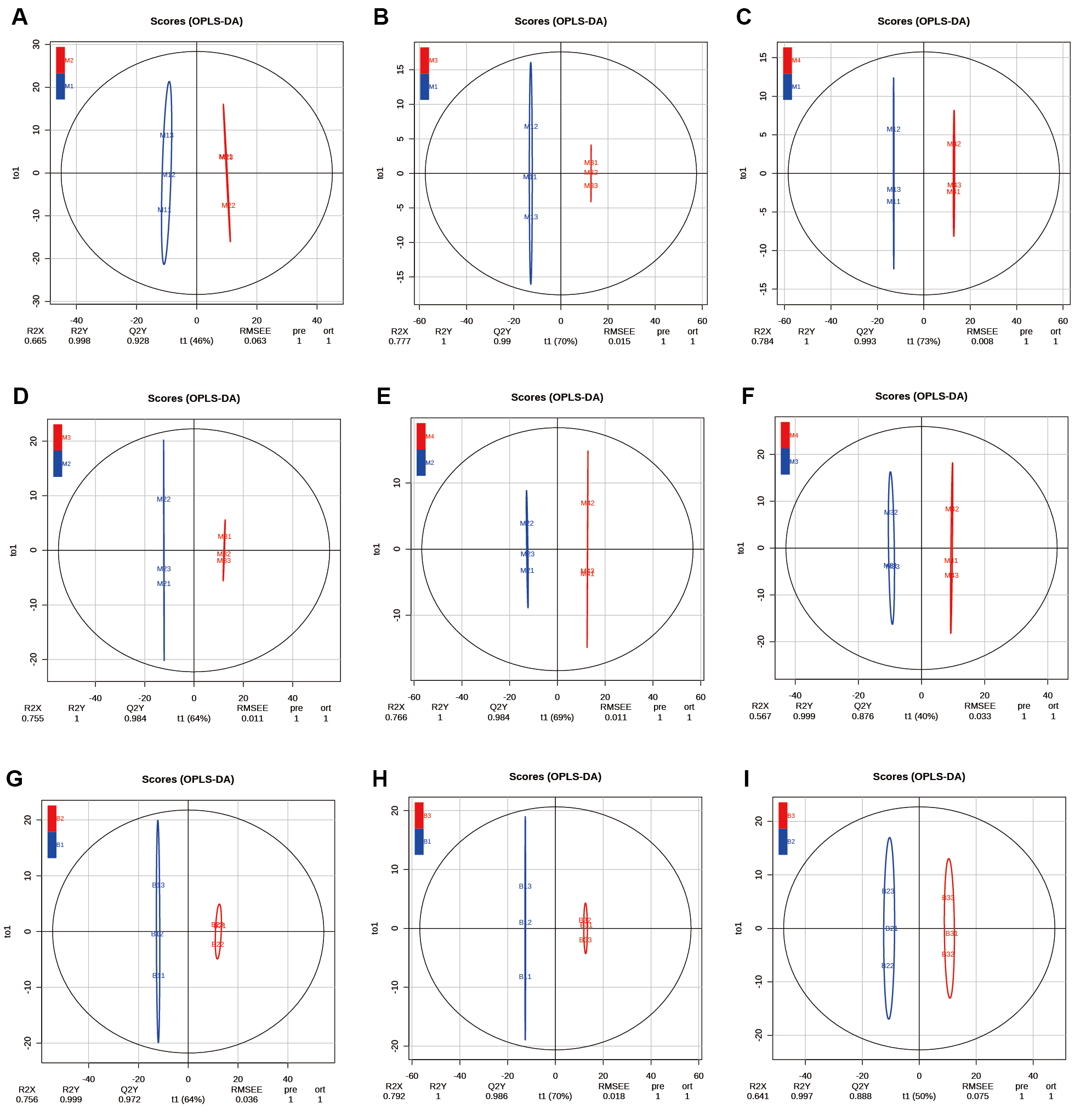

Supplement: Supplementary Figure 1 — OPLS-DA model plots and loading plots for the M1 vs. M2 (A), M1 vs. M3 (B), M1 vs. M4 (C), M2 vs. M3 (D), M2 vs. M4 (E), M3 vs. M4 (F), B1 vs. B2 (G), B1 vs. B3 (H), B2 vs. B3 (I) during fruit development of “MD-2” and “Comte de Paris”. [file Image_1.TIF]
